# Supplementary material for: Cerebellar functional connectivity alteration in individuals with lower limb amputation
Source: PLoS One. 2025 Dec 18;20(12):e0338619. doi: 10.1371/journal.pone.0338619 (PMC12714208; doi:10.1371/journal.pone.0338619)
Supplement: S1 Table — The values in the table represent t-scores for individual functional connections within the S1M1 network, reflecting their statistical significance. A higher absolute t-score indicates a stronger and more statistically robust connection. At the network level, two additional metrics are presented: Mass and Size. Mass represents the total magnitude of connectivity within the network. Size indicates the number of statistically significant connections, providing a measure of the network’s extent. M1 refers to the primary motor area, S1 to the primary sensory area, SMA to the supplementary motor area, Cer to the Cerebellum, i to ipsilateral to amputation, c to contralateral to amputation, S1M1 to sensorimotor network, FDR to false discovery rate correction, and FWE to family wise error correction. (DOCX) [file pone.0338619.s001.docx]

**Supporting material**

###

| **S1 Table. S1M1 network functional connectivity characterization results for the HC group**. | | | | |
| --- | --- | --- | --- | --- |
| Network | Statistic | *p-value* | *p*-FDR | *p*-FWE |
| S1M1 | **Mass** = 4161.49 | 0.000 | 0.000 | 0.000 |
|  | **Size** = 60 | 0.000 | 0.000 | 0.000 |
| Connections |  |  |  |  |
| M1.i - M1.c | T_(25)_ = 14.32 | 0.000 | 0.000 | - |
| M1.i - S1.c | T_(25)_ = 14.25 | 0.000 | 0.000 | - |
| SMA.i - S1.c | T_(25)_ = 13.39 | 0.000 | 0.000 | - |
| M1.i - SMA.i | T_(25)_ = 12.58 | 0.000 | 0.000 | - |
| SMA.i - SMA.c | T_(25)_ = 11.76 | 0.000 | 0.000 | - |
| CerIV-V.i - CerIV-V.c | T_(25)_ = 11.43 | 0.000 | 0.000 | - |
| M1.c - S1.c | T_(25)_ = 10.12 | 0.000 | 0.000 | - |
| M1.c - SMA.i | T_(25)_ = 10.11 | 0.000 | 0.000 | - |
| M1.i - S1.i | T_(25)_ = 10.06 | 0.000 | 0.000 | - |
| SMA.i - S1.i | T_(25)_ = 9.83 | 0.000 | 0.000 | - |
| S1.i - SMA.c | T_(25)_ = 9.45 | 0.000 | 0.000 | - |
| S1.c - S1.i | T_(25)_ = 9.37 | 0.000 | 0.000 | - |
| M1.c - S1.i | T_(25)_ = 8.97 | 0.000 | 0.000 | - |
| CerIV-V.c - CerVI.c | T_(25)_ = 7.51 | 0.000 | 0.000 | - |
| CerIV-V.i - CerVI.c | T_(25)_ = 7.47 | 0.000 | 0.000 | - |
| S1.c - SMA.c | T_(25)_ = 6.86 | 0.000 | 0.000 | - |
| M1.c - SMA.c | T_(25)_ = 6.64 | 0.000 | 0.000 | - |
| M1.i - SMA.c | T_(25)_ = 6.26 | 0.000 | 0.000 | - |
| M1.i - CerIV-V.c | T_(25)_ = 6.15 | 0.000 | 0.000 | - |
| CerVI.c - CerVI.i | T_(25)_ = 5.66 | 0.000 | 0.000 | - |
| M1.c - CerIV-V.c | T_(25)_ = 4.68 | 0.000 | 0.000 | - |
| SMA.i - CerIV-V.c | T_(25)_ = 3.29 | 0.002 | 0.006 | - |
| M1.c - CerIV-V.i | T_(25)_ = 3.14 | 0.004 | 0.008 | - |
| SMA.i - CerIV-V.i | T_(25)_ = 3.11 | 0.004 | 0.018 | - |
| S1.c - CerIV-V.i | T_(25)_ = 3.10 | 0.004 | 0.008 | - |
| S1.i - CerVI.i | T_(25)_ = -2.94 | 0.006 | 0.011 | - |
| M1.i - CerIV-V.i | T_(25)_ = 2.71 | 0.011 | 0.019 | - |
| S1.i - CerIV-V.c | T_(25)_ = 2.37 | 0.025 | 0.041 | - |
| S1.c - CerIV-V.c | T_(25)_ = 2.30 | 0.029 | 0.046 | - |
| SMA.i - CerVI.c | T_(25)_ = 2.29 | 0.030 | 0.046 | - |
| *The values in the table represent* ***t-scores*** *for individual functional connections within the S1M1 network, reflecting their statistical significance. A higher absolute t-score indicates a stronger and more statistically robust connection. At network level, two additional metrics are presented:* ***Mass*** *and* ***Size****. Mass represents the total magnitude of connectivity within the network. Size indicates the number of connections that are statistically significant, providing a measure of the network's extent****. M1*** *refers to primary motor area,* ***S1*** *to primary sensory area,* ***SMA*** *to supplementary motor area,* ***Cer*** *to Cerebellum,* ***i*** *to ipsilateral to amputation,* ***c*** *to contralateral to amputation,* ***S1M1*** *to sensorimotor network,* ***FDR*** *to false discovery rate correction and* ***FWE*** *to family wise error correction.* | | | | |

### 
