# Supplementary material for: Cerebellar functional connectivity alteration in individuals with lower limb amputation
Source: PLoS One. 2025 Dec 18;20(12):e0338619. doi: 10.1371/journal.pone.0338619 (PMC12714208; doi:10.1371/journal.pone.0338619)
Supplement: S2 Table — The values in the table represent t-scores for individual functional connections within the S1M1 network, reflecting their statistical significance. A higher absolute t-score indicates a stronger and more statistically robust connection. At the network level, two additional metrics are presented: Mass and Size. Mass represents the total magnitude of connectivity within the network. Size indicates the number of statistically significant connections, providing a measure of the network’s extent. M1 refers to the primary motor area, S1 to the primary sensory area, SMA to the supplementary motor area, Cer to the Cerebellum, i to ipsilateral to amputation, c to contralateral to amputation, S1M1 to the sensorimotor network, FDR to false discovery rate correction, and FWE to family wise error correction. (DOCX) [file pone.0338619.s002.docx]

**Supporting material**

###

| **S2 Table. S1M1 network functional connectivity characterization results for the AMP group**. | | | | |
| --- | --- | --- | --- | --- |
| Network | Statistic | *p-value* | *p*-FDR | *p*-FWE |
| S1M1 | **Mass** = 3935.79 | 0.000 | 0.000 | 0.000 |
|  | **Size** = 58 | 0.000 | 0.000 | 0.000 |
| Connections |  |  |  |  |
| M1.i - M1.c | T_(25)_ = 20.21 | 0.000 | 0.000 | - |
| M1.i - SMA.i | T_(25)_ = 13.06 | 0.000 | 0.000 | - |
| SMA.i - SMA.c | T_(25)_ = 12.30 | 0.000 | 0.000 | - |
| M1.i - S1.i | T_(25)_ = 12.14 | 0.000 | 0.000 | - |
| S1.c - M1.c | T_(25)_ = 10.47 | 0.000 | 0.000 | - |
| SMA.i - S1.i | T_(25)_ = 10.37 | 0.000 | 0.000 | - |
| CerIV-V.i - CerIV-V.c | T_(25)_ = 10.36 | 0.000 | 0.000 | - |
| S1.c - S1.i | T_(25)_ = 10.16 | 0.000 | 0.000 | - |
| M1.c - S1.i | T_(25)_ = 8.56 | 0.000 | 0.000 | - |
| M1.c - SMA.i | T_(25)_ = 8.55 | 0.000 | 0.000 | - |
| S1.i - SMA.c | T_(25)_ = 8.49 | 0.000 | 0.000 | - |
| M1.i - SMA.c | T_(25)_ = 7.96 | 0.000 | 0.000 | - |
| S1.c - M1.i | T_(25)_ = 7.77 | 0.000 | 0.000 | - |
| S1.c - SMA.i | T_(25)_ = 7.30 | 0.000 | 0.000 | - |
| M1.c - SMA.c | T_(25)_ = 6.33 | 0.000 | 0.000 | - |
| CerVI.c - CerVI.i | T_(25)_ = 6.02 | 0.000 | 0.000 | - |
| S1.c - SMA.c | T_(25)_ = 5.74 | 0.000 | 0.000 | - |
| M1.i - CerVI.c | T_(25)_ = -4.73 | 0.000 | 0.000 | - |
| S1.i - CerVI.i | T_(25)_ = -4.63 | 0.000 | 0.000 | - |
| M1.c - CerVI.c | T_(25)_ = -4.26 | 0.000 | 0.000 | - |
| S1.i - CerVI.c | T_(25)_ = -4.18 | 0.000 | 0.000 | - |
| CerIV-V.c - CerVI.c | T_(25)_ = 4.12 | 0.000 | 0.000 | - |
| M1.c - CerVI.i | T_(25)_ = -3.75 | 0.000 | 0.001 | - |
| SMA.i - CerVI.i | T_(25)_ = -3.42 | 0.002 | 0.004 | - |
| M1.i - CerVI.i | T_(25)_ = -3.08 | 0.004 | 0.008 | - |
| S1.c - CerVI.i | T_(25)_ = -2.81 | 0.009 | 0.016 | - |
| CerIV-V.i - CerVI.c | T_(25)_ = 2.63 | 0.014 | 0.023 | - |
| SMA.c - CerVI.i | T_(25)_ = -2.38 | 0.025 | 0.040 | - |
| S1.c - CerIV-V.i | T_(25)_ = 2.34 | 0.027 | 0.042 | - |
| *The values in the table represent* ***t-scores*** *for individual functional connections within the S1M1 network, reflecting their statistical significance. A higher absolute t-score indicates a stronger and more statistically robust connection. At network level, two additional metrics are presented:* ***Mass*** *and* ***Size****. Mass represents the total magnitude of connectivity within the network. Size indicates the number of connections that are statistically significant, providing a measure of the network's extent****. M1*** *refers to primary motor area,* ***S1*** *to primary sensory area,* ***SMA*** *to supplementary motor area,* ***Cer*** *to Cerebellum,* ***i*** *to ipsilateral to amputation,* ***c*** *to contralateral to amputation,* ***S1M1*** *to sensorimotor network,* ***FDR*** *to false discovery rate correction and* ***FWE*** *to family wise error correction.* | | | | |

### 
