# Supplementary material for: Cerebellar functional connectivity alteration in individuals with lower limb amputation
Source: PLoS One. 2025 Dec 18;20(12):e0338619. doi: 10.1371/journal.pone.0338619 (PMC12714208; doi:10.1371/journal.pone.0338619)
Supplement: Attachment — Submitted filename: Response to reviewers.docx [file pone.0338619.s004.docx]

### **Overall Assessment**

"Cerebellar Modifications of Brain Functional Connectivity in Individuals with Lower Limb Amputation" by Arreguín et al. reports an investigation of differences in functional connectivity between a group of 26 individuals with lower limb amputation, compared to 26 matched healthy control participants, using resting-state functional magnetic resonance imaging. The reported finding of a significant anticorrelation between a spherical region of interest designated as the ipsilateral primary motor cortex and a spherical region of interest designated as the contralateral cerebellar lobule VI may be of some interest.

However, it is not possible to recommend the manuscript for publication as it is currently presented, for the following reasons:

1. The stated conclusions are not supported by the presented results, as most of the reported findings do not reach statistical significance.
2. The finding of a significant anticorrelation does not appear to be consistent with the definition of functional connectivity and therefore requires proper interpretation.
3. The use of the "network-based statistics" (NBS) methodology to calculate only correlations between pairs of regions, rather than broader networks, seems suboptimal.
4. The overall approach—the analytical methodology used and the interpretation of the results—ignores a large body of literature indicating that amputation generally results in "massive functional reorganization in the somatosensory cortex" (see below).
5. The submission does not meet PLOS ONE's data sharing requirement.

### **General Response**

We have carefully considered the observations made on the submitted manuscript and have decided to revise and modify the preprocessing of our analysis. Some of the comments referred to this specific step of the image analysis, and addressing them has led to more precise results regarding the connectivity differences between groups.

Below, we provide a point-by-point response to each comment, detailing the modifications made in the manuscript.

The following modifications were implemented: Initially, during image preprocessing, we performed slice timing correction (STC) after realignment and susceptibility distortion correction using field maps. The modification consisted of performing STC before realignment to prevent any geometric modifications to the images prior to this step (Parker & Razlighi, 2019).

In the original preprocessing, a 6 mm kernel was set for smoothing, considering a 4 mm voxel acquisition. However, we had omitted resampling the images to a 2 mm voxel resolution during normalization to MNI space. We have corrected this by using a 4 mm kernel, following the general recommendation to set a smoothing kernel between 1.5 and 2 times the voxel size (Candemir, 2023; Poldrack, Mumford, & Nichols, 2011).

In the original analysis, subject age was used as a covariate in the calculation of differences. However, we have determined that this is redundant given that the subjects were already matched by age to form the groups. Therefore, the analysis was re-run without this covariate.

As a result of these modifications to the preprocessing and difference analysis, we found similar results. We retained the identification of a significant decrease in the connection between the primary motor region ipsilateral to the amputation and cerebellar lobule 6 contralateral to the amputation (M1.i-CerVI.c). Furthermore, we found a new connection with a significant decrease between the primary sensory region ipsilateral to the amputation and the same cerebellar lobule (S1.i-CerVI.c). Both connections converge on the CerVI.c node, which allows these connections to be considered a subnet of significant connectivity, resulting in a significant decrease in connectivity in the amputation group compared to the non-amputation group. This allows us to discuss changes at the network level and not just a single connection between two nodes within the sensorimotor network.

Finally, the corresponding figures, tables, and text were modified to address the provided observations and analysis changes.

**Major Concerns**

1.Most results fail to reach statistical significance.

Table 3 lists the "differences in functional connectivity between regions of interest (ROI) between [groups]", but only the first item survives correction for multiple comparisons and is, therefore, statistically significant. The others are not.

Consequently, the claims in the abstract that "statistical analysis revealed a decrease in connectivity in the connections…" (note: "connections," plural) and that "the findings… [highlight] a significant decrease in functional connectivity between the contralateral cerebellum and primary sensorimotor areas…" (note: "areas," plural) are not supported by the presented results, because only one interregional connection was found to differ significantly between the groups.

### **Response**

We appreciate this observation and agree that the original manuscript included imprecise claims regarding our findings by using plural nouns (e.g., "connections," "findings"). After updating the image analysis, we identified a significant network component consisting of two connections: one between the ipsilateral primary somatosensory cortex (S1.i) and the contralateral cerebellar lobule VI (CerVI.c), and another between the ipsilateral primary motor cortex (M1.i) and the same cerebellar lobule (CerVI.c). Both of these connections survived false discovery rate correction (p-FDR < 0.05).

Consequently, we have revised the manuscript to more accurately reflect this finding, using the term "**network component**" instead of "connections" to avoid unsupported generalizations. Table 3 has also been updated to reflect these findings.

1.1.- The use of ten regions of interest produces a total of 45 possible interregional connections. Therefore, using a p-level threshold of 0.05, without correction for multiple comparisons, it would be expected that a pure noise analysis would generate an average of 0.05 x 45 = 2.25 apparent but not truly significant differences between the groups. Of course, repeating this thought experiment with a new set of pure noise would produce a similar number of apparent but not truly significant differences between the groups, but it would be overwhelmingly likely to be a different set of interregional correlations; such results would not be expected to be replicated.

### **Response**

We appreciate this observation. Both the original and corrected analyses used a connection-level threshold of a **T-score > 3** and a **False Discovery Rate (FDR) correction** (p-FDR < 0.05). This threshold allows us to detect subtle effects while controlling the probability of including false positives in the final connection-level results, which is appropriate for our study. While the arbitrary choice of this free parameter could affect the analysis's sensitivity, the Network-Based Statistics (NBS) method empirically controls this by performing permutations (1000 simulations by default in CONN). We applied **FDR correction** (p-FDR < 0.05) for between-group comparisons at the connection level and **Family-Wise Error (FWE) correction** (p-FWE < 0.05) for between-group comparisons at the network level. The reported results survived both corrections, leading us to infer that they are not attributable to chance or false positives. These significance thresholds have now been added to the revised Results Table 3 (p. 8).

1.2.- Table 3 in the current manuscript lists one correlation that is statistically significant (because it survives correction for multiple comparisons), plus five more that are not statistically significant. That the authors report five such insignificant correlations, when the expected average number is 2.25, is of little interest. These same findings are graphically represented in Figures 2 and 3; again, most of what is shown here is not statistically significant, and such insignificant findings cannot adequately support any scientific conclusion.

### **Response**

We appreciate the reviewer's observation and agree that the original version included non-significant data that could lead to misinterpretations. In the corrected manuscript, we've removed the non-significant results from Table 3 to present only those that survived the corrections at both the network and connection levels (p-FDR < 0.05 and p-FWE < 0.05).

1.3 Finally, the manuscript title is not supported by the presented results. Since only one "connection" is significantly different between the amputee group and the reference group, the title's reference to "modifications" (plural) is not appropriate.

### **Response**

After the corrections made to the analysis, we found a difference in a network component composed of two connections between three nodes, which converge on the node identified as CerVI.c. In response to the reviewer's recommendation to modify the title, we propose the following to more accurately reflect the findings in the network component: "Cerebellar Functional Connectivity Alteration in Individuals with Lower Limb Amputation."

2.- Anticorrelation is not connectivity.

The statistically significant finding is an anticorrelation, but the negative sign (the finding of a negative correlation coefficient) is neither mentioned nor discussed. Functional connectivity is defined as synchronous neuronal activity at spatially remote locations, which corresponds to correlation. However, the reported finding is an anticorrelation (a correlation coefficient with a negative sign). This does not align with the definition of functional connectivity. Instead, it resembles the well-known temporal anticorrelation of activity in the default mode network with respect to activity in task-positive regions. The finding is of some interest, but its sign is important: the anticorrelation must be presented and discussed as such, not ignored and treated as if it were a positive correlation.
**Response**

We appreciate the reviewer's observation. In the corrected version of the manuscript, we have used the term "anticorrelation" (e.g., "This subnetwork was specifically composed of two anticorrelated connections…," p. 11) when referring to the significant connection found between the evaluated regions.

Additionally, we have included a specific discussion on the meaning of negative correlations in resting-state fMRI studies. We have noted that while functional connectivity has been traditionally associated with positive correlations (Friston, 1993), anticorrelations can also reflect inhibitory neuronal processes or complementary inverse interactions in the hemodynamic response between regions (Goelman et al., 2014).

3. Network-based statistics is questionable when restricted to pairs of regions.

Network-based statistics is analogous to the more familiar and well-established cluster-based statistics. The idea of cluster-based statistics is that instead of testing the null hypothesis at every voxel, we test the null hypothesis for each cluster of potentially active voxels. The advantage is increased power; the disadvantage is that we don't know exactly where in the cluster the activity is; we know that there is activity in the cluster, but not precisely where in the cluster.

Network-based statistics is similar: instead of testing the null hypothesis at every voxel, we test the null hypothesis for each network of voxels (or, more generally, nodes). This yields more power, but at the expense of not knowing exactly where within a "hot" network the "activity" truly resides. We can say that the network is hot (or significant), but not where in the network the activity or significance resides.

What is key in both cases is the use of clusters or networks of substantial size. It would be odd to use cluster-based statistics but to limit the analysis to clusters composed of only two voxels. Yet, unless this reviewer is mistaken, the present manuscript appears to do this with network-based statistics. It appears that only correlations between two regions are considered; these are networks composed of only two nodes. This seems a suboptimal use of network-based statistics.
**Response**

We appreciate the reviewer's observation regarding the use of the NBS method. We would like to clarify that our analysis was not limited to pairs of regions but was performed on the whole functional connectivity matrix of 10 × 10 regions of interest (ROIs), which results in a total of 45 possible connections.

The NBS method (Zalesky et al., 2010) was applied to this entire network to identify subnetworks of connections that, collectively, showed a significant difference between the two groups. The result of this analysis was a significant network component (a subnetwork) composed of three nodes and two connections that survived the strict statistical corrections for type I error at the network level (p-FWE < 0.05) and the connection level (p-FDR < 0.05). This finding is not an artifact of a restricted analysis but the result of the rigorous method we employed.

We believe that the use of NBS was the most appropriate and robust methodological choice for our study for several reasons:

- **Non-parametric analysis:** The NBS method does not assume a normal distribution of the data, which is ideal and safer for our dataset, as it is not large enough to meet parametric assumptions reliably.
- **Control of false positives:** NBS controls the family-wise error rate (FWE) across the entire network, thereby drastically reducing the risk of obtaining false positives compared to analyzing individual connections.
- **Network-level analysis:** Although the resulting subnetwork was small, the fact that the network-level analysis identified it as the only significant component gives it a biological validity and meaning that could not be achieved with a pair-wise correlation analysis.

We hope this clarification is helpful and gives you confidence in the robustness of our methodology. In response to the observation, we modified the method description in the manuscript for a better understanding of the method: "The Network-Based Statistic (NBS) is a non-parametric method designed to identify differences..." (p. 8).

4."Reassignment" has been ignored.

It is well established that "peripheral denervations of large expanses of the sensory epithelium, as a result of limb amputation… can lead to massive functional reorganization in the somatosensory cortex…" (from "Thalamic and Cortical Contributions to Neural Plasticity after Limb Amputation" (Florence et al., 2000)).

Indeed, the introductory section of the present manuscript (p. 10) refers to "…cortical functional reorganization after amputation…"

However, the analysis in the present manuscript ignores such functional reorganization, as it is limited to calculating correlations between spatially smoothed MR signals averaged in ten-mm spheres located at fixed positions in neuroanatomical atlas space. The "massive functional reorganization in the somatosensory cortex" of individuals with the amputation is simply ignored. This approach is not optimal for the analysis of these data. See also the last paragraph of the next point.

### **Response**

We appreciate the reviewer's comment and agree that post-amputation cortical reorganization is a widely documented phenomenon, particularly in the somatosensory cortex contralateral to the amputated limb (Flor et al., 2006; Makin et al., 2013). However, the objective of this study was not to map this reorganization but to evaluate possible alterations in functional connectivity between cortical representations of lower limbs previously identified in other studies on people without amputations. To this end, we selected ROIs in MNI space located in the bilateral motor and somatosensory cortex. We acknowledge that this design does not allow for a direct evaluation of cortical remapping, which has been indicated as a perspective for future studies within the discussion: "It is important to acknowledge that the present analysis did not incorporate potential cortical reorganization prior to the evaluation of functional connectivity, a factor that should be considered in future projects" (p. 15).

5.Data Not Shared.

According to PLOS ONE, data must either be submitted as part of the article (as supplementary information), which is impractical for large image datasets like those used here, or it must be stored in a public repository (such as NITRC). Stating that data are "available" from the corresponding author upon request (as per page 6 of the PDF provided for review) is not adequate.

This reviewer supports PLOS ONE's data-sharing requirement and asks the authors to respect it, generally for the sake of open science, which accelerates scientific progress.

Furthermore, in the present case, given point 4 above, it is anticipated that other investigators might want to re-analyze the data, using, for example, group spatial independent component analysis or multilayer community detection, to characterize intergroup differences in cortical networks. Instead of assuming that cortical representations are fixed—despite the extensive literature on "massive functional reorganization in the somatosensory cortex" of individuals with amputation—and comparing correlations between specific (atlas-based) spherical regions of interest, as the authors have done here, other investigators might be interested in evaluating the spatial distribution and network strength (using spatial independent component analysis or other algorithms) and/or network graph-theoretic characteristics, such as segregation and recruitment coefficients (using multilayer community detection or other algorithms). This makes data sharing even more important.

### **Response**

The authors are in the process of uploading the data to a public repository. A request has already been submitted in accordance with the site's guidelines. We will provide an update as soon as the data becomes available.

**OTHER CONCERNS**

**Reviewer 1**

6.Why were "connectivity differences... controlling for the influence of age?" (pp. 16 and 18) evaluated? What is the justification for this? Were the study data also analyzed without controlling for age?

### **Response**

Thank you for your observation. In the corrected version of the manuscript, and after making the necessary image processing adjustments, we removed age as a covariate from the functional connectivity analysis. The groups were already matched by age during participant selection, and no statistically significant differences were found for this variable (p > 0.05). Therefore, statistically controlling for age as a covariate would have resulted in overfitting in the analysis.

7.There is some concern about the procedure of flipping images of right-side amputee participants ("the images from participants with a right-side amputation were subjected to a hemispheric flip across the mid-sagittal midline," p. 14), since none of the pairs of regions of interest that were used are mirror-symmetric with respect to the midline (according to Table 1). This suggests that such a flip could bias the results.

### **Response**

We appreciate the reviewer's observation regarding the potential introduction of bias from the hemispheric flipping procedure for participants with a right-sided amputation. This flip was performed to standardize hemispheric laterality among participants, thereby allowing for a consistent analysis of the hemisphere responsible for processing the amputated limb, regardless of the amputation side. It is important to note that the regions of interest (ROIs) were not selected based on anatomical symmetry, but rather on their functional relevance in the motor and sensory processing of the lower limbs, as established by previous neuroimaging activation studies (Jaeger et al., 2014; Newton et al., 2008). Therefore, the flip does not aim to force anatomical symmetry between regions, but rather to analyze the functional representation proposed by these studies to enable comparisons between the groups.

8.Slice timing correction appears to have been performed late in the pipeline (p. 14), which is concerning. Once the images have been coregistered, realigned, and warped, performing slice timing correction is problematic, as the data no longer respects the acquisition geometry.

### **Response**

We appreciate the reviewer's observation and agree that the preprocessing order can affect data integrity. Consequently, we adjusted our pipeline to apply slice timing correction (STC) before realignment and susceptibility distortion correction. This ensures that STC is performed on data that still respects the acquisition geometry. This change, reflected in the corrected manuscript, aligns our analysis with best practices in functional connectivity studies and improves the robustness of our results. "This pipeline generated slice-timing correction (STC), voxel displacement maps for motion and susceptibility-induced distortion corrections through fieldmaps, outliers detection, indirect segmentation, normalization to the MNI space, and subsequent smoothing."

9. "The use of NBS, based on permutation tests, proved reliable and robust as an alternative…" (p. 19). This statement raises two concerns: A. No results presented in the current manuscript support the claim that NBS "proved reliable and robust" in this study. B. It is unclear what "alternative" means here: what is NBS an alternative to? See also point 3 above.

### **Response**

We appreciate the reviewer's point. We recognize that the claim about the reliability and robustness of the NBS method was not supported by direct evidence in our results, and that the use of the term "alternative" was ambiguous. Therefore, these phrases were removed from the corrected manuscript to avoid unjustified interpretations.

10.The supplementary table indicates that all participants in both groups were right-handed; this should be clearly stated in the manuscript.

### **Response**

In the corrected manuscript, we indicate that all participants in both groups were right-handed: "Handedness was assessed for all participants, and it was confirmed that every member of both groups was right-handed using the Edinburgh Handedness Inventory" (p. 7).

11."the Pearson correlation test" (p. 15). The Pearson correlation coefficient is not a "test."

### **Response**

We appreciate the reviewer's observation. We agree that the Pearson correlation coefficient should not be described as a "test." In the corrected manuscript, we have replaced the phrase with a more accurate one: "Functional connectivity strength was represented by Fisher-transformed bivariate correlation coefficients from a general linear model..." (p. 8), to properly reflect the statistical procedure used.

12."ROI-ROI connectivity maps were calculated for each participant..." (p. 15). These were matrices, not "maps."

### **Response**

We appreciate the reviewer's observation. In the corrected manuscript, we have replaced "maps" with the more appropriate term: "The average time series for each ROI were extracted, and ROI-ROI connectivity matrices (10 x 10) were generated" (p. 8).

**Reviewer 2**

The study investigates alterations in functional connectivity within the sensorimotor network of 26 individuals with lower limb amputation using resting-state fMRI. The results show a decrease in connectivity between the contralateral cerebellum and primary sensorimotor areas, which suggests neuroadaptive changes following amputation. However, the reviewer has the following main concerns regarding the study's methodology, data interpretation, and statistical analysis:

1.The manuscript does not provide a power analysis or a justification for the sample size of 26 amputees. Given the complexity of functional connectivity analyses, this small sample may lack statistical power.

**Response**

Thank you for your observation. While a formal power analysis was not conducted, our sample size of 26 participants per group was based on widely used recommendations for fMRI studies aiming to detect large effects with 80% statistical power at an alpha level of 0.05.

Furthermore, in ROI-ROI connectivity studies, the Network-Based Statistics (NBS) method is known to increase sensitivity for detecting network-level differences even in small samples. We also took into account recent reports (Szucs & Ioannidis, 2020), which indicate that our sample size is larger than the median observed in similar fMRI studies.

2.The study does not adequately control for potential confounding factors, such as years of education, prosthetic use, and the duration of the amputation. These factors could significantly affect functional connectivity patterns.

### **Response**

We appreciate the comment regarding the potential variability of the results linked to the sampling characteristics of this study. Unfortunately, in human studies, it isn't easy to control for all the aspects you mentioned, especially in a hard-to-reach population like the one we studied.

However, our sampling aimed to control this variability as much as possible by reducing the time elapsed since the participants' amputations. We also matched both groups by age and included participants with no more than 36 months post-amputation to obtain the most homogeneous sample possible.

We recognize that factors such as education level, sex, and prosthetic use can influence functional connectivity patterns. Therefore, the need for future longitudinal studies with a larger sample size and the inclusion of these covariates is mentioned in the corrected manuscript. We believe that despite the possible variability linked to these factors, the results obtained in the present study are supported by an adequate non-parametric analysis methodology, which lends credibility to the observations included in the discussion.

3.The selection of 10 ROIs lacks a clear justification. Why were these specific coordinates chosen, and how do they compare with previous studies? More details are needed on how these ROIs represent the sensorimotor network.
Response

Thank you for your comment. The selection of the 10 ROIs is based on their key role within the sensorimotor network involved in the motor control of the lower extremities. The regions included (M1, S1, SMA, and cerebellar lobules IV, V, and VI) have been consistently reported as active areas in studies of specific motor tasks, such as ankle dorsiflexion, hip extension, and isometric quadriceps contractions (Jaeger et al., 2014; Newton et al., 2008; Stoodley & Schmahmann, 2009).

The MNI coordinates were taken from these works and reflect reproducible activations in healthy subjects during passive and active motor tasks. This approach, based on previous literature, is widely used to evaluate functional connectivity in regions involved in limb control. The ROIs were defined to explore changes in functionally relevant regions, including preserved areas that control the non-amputated limb. A detailed description of each ROI and its coordinates is included in Table 1 of the manuscript.

4.The analysis reports significant findings at an uncorrected p < 0.05, which is problematic given the number of comparisons performed. A more stringent correction, such as Bonferroni or permutation testing, should be applied.

### **Response**

We appreciate your observation. After reviewing the manuscript, we corrected a wording error. The functional connectivity analysis was performed using the Network-Based Statistics (NBS) method, which incorporates permutation tests (1000 simulations in the CONN software) to control for multiple comparisons at the network level. In this analysis, significance was evaluated with an FDR correction (p<0.05) at the individual connection level and an FWE correction (p<0.05) for mass and size at the network component level. The corrected manuscript has been updated to reflect this with greater clarity.

5.The manuscript attributes reductions in functional connectivity to neuroadaptive processes without considering alternative explanations, such as motion artifacts or scanner-related issues. Additional validation is required.

Response

We appreciate this comment. We acknowledge that motion artifacts and scanner-related noise can influence functional connectivity estimations. To minimize these effects, we applied quality controls during both the image acquisition and preprocessing phases.

During acquisition, we used a standardized protocol recommended for resting-state connectivity studies, which included head stabilization and continuous motion monitoring with participants focusing on a fixation cross. In the preprocessing pipeline, we used conservative exclusion criteria with the ART toolbox (implemented in CONN), discarding volumes with framewise displacements (FWD) greater than 2.5 mm or changes in the global BOLD signal percentage exceeding 3 standard deviations (Ashburner & Friston, 2005).

6.A cross-sectional design limits interpretations. The manuscript must acknowledge that without pre- and post-amputation comparisons, it cannot definitively attribute changes to the amputation itself.

### **Response**

We appreciate this comment. We have incorporated and discussed this limitation in the corrected manuscript: "It is essential to acknowledge that the cross-sectional design of this study is an inherent limitation..." (p. 14). While a longitudinal approach with pre- and post-amputation assessments would offer greater robustness, its implementation was not feasible in our context due to the urgent medical nature of the amputation event, which makes it difficult to recruit participants prior to amputation. Therefore, we opted for a comparison with a healthy control group, while recognizing that this strategy does not completely eliminate potential biases emphasizing the need for longitudinal studies in the future.

7.Some statistical tests, such as those used for connectivity differences, are described in insufficient detail. The authors should clarify whether parametric or non-parametric methods were used consistently.
**Response**

We appreciate this observation. In the corrected manuscript, we have clarified that the Network-Based Statistics (NBS) method was used for the analysis of functional connectivity differences. This is a non-parametric approach based on permutations. The method applies a generalized linear model with independent two-sample t-tests, using connectivity matrices with r to Fisher's Z-transformed values. Through 1000 permutations, a null distribution is generated to compare the observed differences, controlling for multiple comparisons using the FDR method at the connection level and the FWE method at the network component level. This procedure was consistently employed in the reported connectivity analysis.

8.While the findings suggest changes in connectivity, the manuscript does not link them to functional or behavioral outcomes. Claims regarding clinical significance should be supported by direct evidence.

### **Response**

We appreciate this observation. The claims relating the connectivity changes to functional or behavioral outcomes have been removed in the corrected manuscript. Instead, the need for future studies focused on this relationship with direct evidence is discussed: "To understand the trajectory of these neurofunctional adaptations and their association with the amputation experience, longitudinal studies incorporating pre and post-rehabilitation assessments, including clinical and behavioral evaluations, will be essential" (p. 15).

9.Head motion is a significant confounding factor in resting-state fMRI. The manuscript does not report the framewise displacement values or whether high-motion participants were excluded.

### **Response**

We appreciate your observation. In the present study, strategies were implemented to mitigate the influence of motion on fMRI data. During preprocessing, thresholds were applied to identify and correct for images with excessive motion using the ART toolbox included in the CONN software (Ashburner & Friston, 2005). Specifically, images were identified as outliers if they presented framewise displacements (FWD) greater than 2.5 mm or a change in the global BOLD signal exceeding 3 standard deviations. Although the exact number of discarded images was not recorded, we can confirm that no participants were excluded from the analysis due to excessive motion. This information has been incorporated into the "Image Preprocessing" section of the corrected manuscript's methods.

10.The manuscript states that 5 mm ROIs were used, but does not justify this choice. Furthermore, a 6 mm smoothing kernel may dilute regional specificity, which affects the results.
**Response**

We appreciate the comment. In the corrected manuscript, we mention that a spatial smoothing of FWHM = 4 mm was applied (not 6 mm, as mentioned in the original manuscript), following resampling to 2 mm isotropic. At this resolution, the use of spherical ROIs with a 5 mm radius (10 mm diameter) is appropriate, as it allows for the capture of a representative BOLD signal with a sufficient number of voxels, minimizing physiological noise without including irrelevant neighboring regions. This decision is based on both anatomical and methodological criteria and is supported by previous literature on resting-state functional connectivity studies (Poldrack et al., 2011; Biswal et al., 1995). The coordinate assignment maximizes the anatomical distance between ROIs to avoid overlap and preserve functional specificity.

11.The manuscript reports on hemispheric flipping in right-sided amputees but does not validate whether this step preserves functional connectivity patterns.

### **Response**

The hemispheric flipping procedure was used to anatomically homologize the hemispheres ipsilateral and contralateral to the amputation, standardizing cortical representations across participants with either left or right amputation. This decision is supported by evidence that the sensorimotor network shows interhemispheric functional symmetry, particularly in the resting state (Jin et al. 2020). These studies have identified homotopic connectivity between bilateral motor and somatosensory regions, which justifies the use of flipping to homologize functional laterality. While a comparative connectivity analysis before and after the flip was not performed in the present study, this methodological choice aligns with practices used in studies of cortical reorganization in unilateral lesions, where a common functional reference is sought.

12.Some references (e.g., Koziol et al., 2014 on cognition and the cerebellum) do not directly support the claims made and should be replaced with more relevant citations.

### **Response**

We appreciate the observation. We agree that the citation to Koziol et al. (2014), while relevant as a theoretical review, does not directly support the specific claims in the manuscript. In the corrected version, this reference has been replaced and supplemented with functional activation meta-analysis studies that provide more pertinent evidence regarding the cerebellum's sensorimotor role (Dijkstra BW., 2020; Hardwick RM., 2013; Stoodley CJ., 2008; Thach WT., 2004).

13.The TR and TE values are provided, but essential details such as the field of view, voxel size, and number of volumes per series are not clearly reported.

### **Response**

We appreciate the observation. To enhance clarity in the corrected manuscript, we've included the acquisition parameters for the functional, anatomical, and inverse phase-encoding images: "Resting-state functional images were obtained through an axial echo-planar imaging (EPI-GR) sequence with the following parameters: 300 volumes, TR = 2000 ms, TE = 40 ms, flip angle = 90º, matrix = 64 x 64, slice thickness = 4 mm, voxel size = 4 x 4 x 4 mm and FOV = 256 x 256 mm…" (p. 5).

14.The manuscript presents connectivity findings in Figures 2 and 3 but fails to provide complete statistical details in the figure legends (e.g., exact p-values for non-significant findings).

### **Response**

We appreciate the observation. In the revised version of the manuscript, we have updated the figures to represent the results more accurately. We have incorporated complete statistical details into the legend of Figure 2, including the exact p-values corresponding to the significant findings at both the network component and individual connection levels. Additionally, Figure 3 now only displays the significant results. In line with another reviewer's recommendations, we have chosen not to include non-significant findings to avoid misinterpretations, focusing exclusively on reporting the statistically relevant effects.

15.The introduction presents a general hypothesis that connectivity should decrease in amputees but does not specify which regions or connections are predicted to be affected.

### **Response**

We appreciate the observation. Indeed, in the original version of the manuscript, the general hypothesis proposed a decrease in functional connectivity in people with amputation, but without specifying the level of analysis.

In the corrected version, the hypothesis was reformulated to explicitly align with the methodological approach used (NBS), indicating that a network component with reduced functional connectivity would be expected in the amputation group compared to the control group. Given that the study's objective was not to identify specific connections with significant differences but to evaluate differences at the level of topologically organized subnetworks, we believe this reformulation improves conceptual and methodological precision without compromising the scope of the study.

**REFERENCES**

Aguirre, D. (2011, March 23). Tamaño muestra para estudios de RMF en 1_5 T - Daniel Aguirre. <https://www.researchgate.net/publication/258208521>

Ashburner, J., & Friston, K. J. (2005). Unified segmentation. NeuroImage, 26(3), 839–851. <https://doi.org/10.1016/j.neuroimage.2005.02.018>

Biswal, B., Zerrin Yetkin, F., Haughton, V. M., & Hyde, J. S. (1995). Functional connectivity in the motor cortex of resting human brain using echo-planar mri. Magnetic Resonance in Medicine, 34(4), 537–541. <https://doi.org/10.1002/mrm.1910340409>

Candemir C. (2023). Spatial Smoothing Effect on Group-Level Functional Connectivity during Resting and Task-Based fMRI. Sensors (Basel, Switzerland), 23(13), 5866.<https://doi.org/10.3390/s23135866>

Dijkstra, B. W., Bekkers, E. M. J., Gilat, M., de Rond, V., Hardwick, R. M., & Nieuwboer, A. (2020). Functional neuroimaging of human postural control: A systematic review with meta-analysis. Neuroscience and biobehavioral reviews, 115, 351–362. <https://doi.org/10.1016/j.neubiorev.2020.04.028>

Flor, H., Nikolajsen, L., & Staehelin Jensen, T. (2006). Phantom limb pain: a case of maladaptive CNS plasticity? Nature Reviews Neuroscience, 7(11), 873–881. <https://doi.org/10.1038/nrn1991>

Florence, S. L., Hackett, T. A., & Strata, F. (2000). Thalamic and cortical contributions to neural plasticity after limb amputation. Journal of Neurophysiology, 83(5), 3154–3159.<https://doi.org/10.1152/jn.2000.83.5.3154>

Friston, K. J., Frith, C. D., Liddle, P. F., & Frackowiak, R. S. J. (1993). Functional connectivity: The principal-component analysis of large (PET) data sets. Journal of Cerebral Blood Flow & Metabolism, 13(1), 5–14.<https://doi.org/10.1038/jcbfm.1993.4>

Foell, J., Bekrater-Bodmann, R., Diers, M., & Flor, H. (2016). Mirror therapy for phantom limb pain: Brain changes and the role of body representation. Brain, 139(Pt 8), 2289–2303.<https://doi.org/10.1093/brain/aww109>

Goelman, G., Gordon, N., Bonne, O. (2014). Maximizing Negative Correlations in Resting-State Functional Connectivity MRI by Time-Lag. PLoSONE 9(11):e111554. doi: 10.1371/journal.pone.0111554.

Hardwick, R. M., Rottschy, C., Miall, R. C., & Eickhoff, S. B. (2013). A quantitative meta-analysis and review of motor learning in the human brain. NeuroImage, 67, 283–297. <https://doi.org/10.1016/j.neuroimage.2012.11.020>

Jaeger, L., Marchal-Crespo, L., Wolf, P., Riener, R., Michels, L., & Kollias, S. (2014). Brain activation associated with active and passive lower limb stepping. Frontiers in Human Neuroscience, 8. <https://doi.org/10.3389/fnhum.2014.00828>

Jin, X., Liang, X., & Gong, G. (2020). Functional Integration Between the Two Brain Hemispheres: Evidence From the Homotopic Functional Connectivity Under Resting State. Frontiers in Neuroscience, 14. <https://doi.org/10.3389/fnins.2020.00932>

Makin, T. R., Filippini, N., Duff, E. P., Henderson Slater, D., Tracey, I., & Johansen-Berg, H. (2015). Network-level reorganisation of functional connectivity following arm amputation. *NeuroImage*, *114*, 217–225.<https://doi.org/10.1016/j.neuroimage.2015.02.067>

Newton, J. M., Dong, Y., Hidler, J., Plummer-D’Amato, P., Marehbian, J., Albistegui-DuBois, R. M., Woods, R. P., & Dobkin, B. H. (2008). Reliable assessment of lower limb motor representations with fMRI: Use of a novel MR compatible device for real-time monitoring of ankle, knee and hip torques. NeuroImage, 43(1), 136–146. <https://doi.org/10.1016/j.neuroimage.2008.07.001>

Parker, D. B., & Razlighi, Q. R. (2019). The Benefit of Slice Timing Correction in Common fMRI Preprocessing Pipelines. Frontiers in Neuroscience, 13, 821.<https://doi.org/10.3389/fnins.2019.00821>

Poldrack, R. A., Mumford, J. A., & Nichols, T. E. (2011). Handbook of Functional MRI Data Analysis. Cambridge University Press.

Stoodley, C. J., & Schmahmann, J. D. (2009). Functional topography in the human cerebellum: A meta-analysis of neuroimaging studies. NeuroImage, 44(2), 489–501.<https://doi.org/10.1016/j.neuroimage.2008.08.039>

Szucs, D., & Ioannidis, J. P. A. (2020). Sample size evolution in neuroimaging research: An evaluation of highly-cited studies (1990-2012) and of latest practices (2017-2018) in high-impact journals. NeuroImage, 221, 117164.<https://doi.org/10.1016/j.neuroimage.2020.117164>

Thach, W. T., & Bastian, A. J. (2004). Role of the cerebellum in the control and adaptation of gait in health and disease. Progress in brain research, 143, 353–366. <https://doi.org/10.1016/s0079-6123(03)43034-3>

Zalesky, A., Fornito, A., & Bullmore, E. T. (2010). Network-based statistic: Identifying differences in brain networks. NeuroImage, 53(4), 1197–1207. 1<https://doi.org/10.1016/j.neuroimage.2010.06.041>.
